# Outcome of intravitreal bevacizumab injection without pre and postoperative antibiotics

**DOI:** 10.1186/s12886-020-01420-1

**Published:** 2020-04-15

**Authors:** Ruchi Shrestha, Pratap Karki, Sagun Narayan Joshi

**Affiliations:** 1 Reiyukai Eiko Masunaga Eye Hospital, Kavre, Banepa, Nepal; 2 Department of Retina B. P Koirala Lions Centre for Ophthalmic Studies (BPKLCOS), Maharajgunj, Kathmandu, Nepal; 3 Department of Retina, B.PKoirala Lions Centre for Ophthalmic Studies (BPKLCOS), Maharajgunj, Kathmandu, Nepal

**Keywords:** Bevacizumab, Pre-operative antibiotics, Post-operative antibiotics, Endophthalmitis

## Abstract

**Backgound:**

Intravitreal injections are the most common treatment modality for several retinal pathologies. Despite endophthalmitis being the most feared complication, antibioprophylaxis remains controversial in intravitreal injections.

**Methods:**

This was a retrospective study done for a period of 2 years from 1st January 2017 to 31st December 2018 in B. P Koirala Lions Centre for Ophthalmic Studies (BPKLCOS) among patients receiving intravitreal bevacizumab. The intravitreal injection was given by a single surgeon. It included 503 eyes which received intravitreal bevacizumab over a period of 2 years without pre and postoperative antibiotics.

**Results:**

Out of 503 eyes studied over a period of 2 years without antibiotic prophylaxis the rate of endophthalmitis was 0.0019% which is very low compared to the other studies with rate of endophthalmitis between 0.019–0.09%.

**Conclusion:**

The risk of endophthalmitis was low even without pre/post-operative antibiotics. Intravitreal injection can be given safely without pre-operative and post-operative antibiotics. Trial Registration not applicable as it is a retrospective study.

## Background

The normal flora of the eye comprise of mainly bacteria which do not cause infection in normal conditions but can be a main source of infection after ocular surgery, trauma or in immune compromised. The ranges of these microorganisms vary with age, sex and geographical distribution. Therefore it is very important for the ophthalmologist to know the ocular normal flora before giving prophylactic antibiotics and treating infections [1].

Intravitreal injections are the most common treatment modality for several retinal pathologies [2]. Repeated doses for longer duration are often required for patients receiving anti-Vascular Endothelial Growth Factor (VEGF) injection therapy for retinal disease. The overuse of antibiotics could possibly create and proliferate: resistant strains, increase drug costs and the chances of possible adverse reactions to the drugs administered [3].

Antibiotic prophylaxis remains controversial in intravitreal injections although endophthalmitis is the most feared complication. Repeated use of antibiotic prophylaxis during monthly intravitreal injections promotes resistance and virulence of conjunctival flora even with low doses and short duration [4]. The rate of endophthalmitis in intravitreal injection is very low but the associated visual morbidity is often devastating [5]. Increased bacterial resistances to pre-operative injection of antibiotics with monthly repeated injection have been described [6].

The aim of the study is to evaluate the outcome of intravitreal bevacizumab injection without pre and post-operative antibiotics.

## Methods

This is a retrospective study done for a period of 2 years from 1st January 2017 to December 31st 2018 in BPKLCOS, Kathmandu among patients receiving intravitreal Bevacizumab. The intravitreal injection was given by a single surgeon.

This study included 503 consecutive eyes which received intravitreal Bevacizumab over a period of 2 years without preoperative and postoperative antibiotics. Intravitreal Bevacizumab was given under aseptic precautions in the operating room. All patients were painted with 5% povidone iodine before injection and allowed to stand for 10 mins. Topical povidone iodine was applied before starting intravitreal injection and allowed to stay in conjunctival sac for at least 30 s. The surgeon used sterile gloves, gown, cap and surgical face mask, and the patients made use of the surgical gowns and cap.

While the patient was asked to look down, the upper lid was elevated, the superior bulbar conjunctiva was exposed and the eyelashes were completely covered. In phakic patients, the injection site was 4 mm and in pseudophakic 3.5 mm posterior to the limbus. The needle was directed toward the center of the vitreous cavity.

Insulin syringe (31Gauze) was used to inject 1.25 mg/0.05 ml of Bevacizumab. Cotton tipped applicator soaked with 5% povidone iodine was applied after needle was withdrawn. Each patient was given post-operative instructions and forewarned of the alarming signs of endophthalmitis, such as the ocular pain, decreased vision, and lid edema. Follow up was after 3 weeks.

## Results

The mean age of patients was 59.62 years. The minimum age being 19 years /and the maximum age 91 years. There were 308 males and 195 females in this study. Two hundred sixty-four were right eye and 239 were left eye.

The indications of intravitreal Bevacizumab (Table 1) were diabetic macular edema (36.58%), branch retinal vein occlusion (BRVO) with macular edema (36.18%), central retinal vein occlusion (CRVO) with macular edema (11.92%), choroidal neovascular membrane (CNVM) (14.51%), central serous retinopathy (CSR) (0.39%) and uveitic macular edema (0.39%).

**Table 1:** Indications of bevacizumab injection and the number/percentage of patients involved

Indications of bevacizumab	Number /percentage of patients
Diabetic macular edema	184 (36.58%)
CRVO with macular edema	60 (11.92%)
BRVO with macular edema	182 (36.18%)
CNVM	73 (14.51%)
CSR	2 (0.39%)
Uveitic macular edema	2 (0.39%)

Out of 503 eyes, studied over a period of 2 years without antibiotic prophylaxis the rate of endophthalmitis was 0.0019% ie: 1 eye out of 503 presented with endophthalmitis.

The patient who developed endophthalmitis was a case of uncontrolled diabetes mellitus for 12 years. The patient was 65 years old male. The patient developed endophthalmitis within 2 days of intravitreal bevacizumab injection in one eye. The best corrected visual acuity dropped from 6/18 to perception of light. Intravitreal vancomycin and amikacin was given but his visual acuity didn’t improve. Core vitrectomy was performed along with intravitreal antibiotics but the patient ended up with no perception of light within 1 month.

## Discussion

Intravitreal injection can be given safely without pre/post-operative antibiotics with a low risk of endophthalmitis in this study. This finding could potentially eliminate an unnecessary intervention that is likely harmful to patients and minimize the risk of antibiotic resistance and virulence of conjunctival flora.

The mean age of patients in our study was 59.62 years. The minimum age being 19 years /and the maximum age 91 years. Two hundred sixty-four were right eye and 239 were left eye. Similar to the study done by Afarid M et al. where the mean age (± SD) of the patients was 61.48 (± 11.21) years. Out of 359 patients, 141(39.3%) were men and 218 (60.7%) were women in contrast to our study [7]. There were 308 males and 195 females in our study.

The indications of intravitreal injection in our study were diabetic macular edema, CRVO with macular edema, BRVO with macular edema, choroidal neovascular membrane, central serous retinopathy and uveitic macular edema which was similar to study done by Mohammad et al. [8].

Fluoroquinolones are the most commonly used post-injection prophylactic antibiotics in patients due to their broad spectrum and high penetration. Several studies have demonstrated substantial levels of resistance to third- and fourth-generation fluoroquinolones, as well as multi-drug resistance in patients treated with topical antibiotics after multiple intravitreal injection [8, 9].

Many ophthalmologists continue to recommend a multiday course of topical antibiotic use before and after intravitreal injection due to lack of data to support reduction of endophthalmitis through use of antibiotics. Strict rules of asepsis remain the only evidence-based support for prophylaxis of endophthalmitis. Therefore, antibiotics should be prescribed only in exceptional cases such as immunosuppression or fragile conjunctiva. International guidelines surrounding the use of antibiotics in intravitreal injections should be generated [4].

Povidone Iodine or “half strength” povidone-iodine is routinely used in ophthalmic surgery due to its broad spectrum antimicrobial activity, low incidence of microorganism resistance, cost-effectiveness, and wide availability [10]. In a study reported by Diabetic Retinopathy Clinical Research Network (DRCR.net), 3123 eyes received 28,786 intravitreous injections, usually with povidone-iodine preparation. However, a total of 13 injections in 2 participants were administered without antiseptic and both participants developed endophthalmitis in 1 eye each. This was 15% risk of endophthalmitis per injection. 100% of the risk subjects developed endophthalmitis during the short duration of the treatment [11] The omission of topical antiseptic is associated with significantly higher rates of endophthalmitis.

The rate of endophthalmitis without the use of pre and post injection antibiotics in our study was very low ie 0.0019%. Similarly another study by Benoist showed the incidence of endophthalmitis with antibiotic use was 0.052% versus 0.048% without antibiotic use [4]. Muhammad et al. showed in their study that the use of antibiotics after intravitreal Bevacizumab injection does not make any difference for the prevention of postoperative endophthalmitis. Out of 620 injections given in 480 eyes, 310 were control group without any post-injection medicine and 310 were cases who were given post-injection medicine. No case of proven or suspected endophthalmitis was identified, corresponding to a risk of 0% per injection [12]. Bhatt et al. in their study found the rate of endophthalmitis post injection antibiotics were 0.22% versus not receiving antibiotics were 0.20% The rate of endophthalmitis after intravitreal injections administered in a clinical practice setting when aseptic technique is used is similar with or without the use of post-injection antibiotics [13].

In Endophthalmitis Vitrectomy Study, diabetic patients showed more virulent microorganisms and a higher proportion of Gram negatives and less probability of presenting negative cultures 19 [6]. The risk factors for endophthalmitis in diabetes melllitus are Insulin Dependent Diabetes Mellitus, old age, immunosuppression and most infections arouse from own flora [12]. Although no large-scale study has looked exclusively at subjects with diabetes and endophthalmitis, analysis of the subgroup of patients with diabetes in studies comprising both (patients with diabetes and patients without) suggest that virulence is worse in the former group, growth of organisms is faster and a more aggressive treatment bares a better result [14]. The only patient who developed endophthalmitis in our study was a 64 years old male patient with uncontrolled diabetes mellitus.

The standardized Diabetic Retinopathy Clinical Research Network (DRCR.net) intra vitreous injection protocol requires the application of topical anesthetic, the use of a sterile eyelid speculum, and the application of topical povidone-iodine to the conjunctiva. The protocol does not require but allows topical antibiotics prior to, on the day of, or after the injection. The results of DRCR.net study by Bhavsar et al. were that the rates of endophthalmitis by antibiotic use were 0.11% versus 0.03% without antibiotic use [6].

P.ET.Lau et al. recommended: povidone iodine antisepsis, eyelid retraction with speculum, prevention of droplet spread via masks, adhesive drapes and reduced talking, and subconjunctival anesthetic with lidocaine base agent. The omission of prophylactic topical antibiotics seems justified by the existing literature; however prospective trials are lacking [5].

Recent studies have indicated that the use of topical antibiotics could increase resistance to some antibiotics like fluoroquinolones by affecting the conjunctival and nasopharyngeal flora. Moreover, increasing the proportion of resistant bacteria on the ocular surface increases the risk of developing antibiotic-resistant infections that are difficult to treat. Ocular surface preparation for intravitreal injection using povidone-iodine 5% alone in the absence of post injection topical antibiotics does not appear to promote bacterial resistance or a discernible change in conjunctival flora [15].

Grzybowski et al. recommended expert consensus on intravitreal injections. The topical administration of 5% povidone-iodine over at least 30 s into the conjunctival sac is recommended. It doesn’t recommend the use of perioperative antibiotics for intravitreal injection as in our study [16].

Recommendation from 2014 expert panel came to the consensus that the most important aspects of the antiseptic technique include the use of conjunctival 5% povidone-iodine, avoiding lash or lid touch to the site of injection following the povidone-iodine, and the use of surgical masks or decreased talking during the procedure. It doesn’t recommend the use of antibiotics for intravitreal injection [17] which strongly supports our study.

## Conclusions

The risk of endophthalmitis was low even without pre/post-operative antibiotics. Intravitreal injection can be given safely without the use of antibiotics. The purpose of his study is to minimize the overuse of antibiotics. We ultimately believe that the decision to use antibiotics in the prophylactic period depends on individual ophthalmologists.

## Data Availability

Datasets generated during and/or analyzed during the current study are available from the corresponding author on reasonable request.
